# Efficacy and safety of Gushen Antai Pills combined with dydrogesterone in the treatment of threatened miscarriage: a systematic review and meta-analysis

**DOI:** 10.3389/fphar.2023.1138968

**Published:** 2023-06-02

**Authors:** Kai Chen, Xiaoxia Liu, Xianhua Meng, Hui Li, Chenchen Yang, Xiaohong Wang

**Affiliations:** Department of Obstetrics and Gynecology, Jinan City People’s Hospital, People’s Hospital Affiliated to Shandong First Medical University, Jinan, Shandong, China

**Keywords:** threatened miscarriage, Gushen Antai Pills, dydrogesterone, complementary therapy, meta-analysis

## Abstract

**Background:** Despite some progress has been made in the pathogenesis and treatment of threatened miscarriage (TM), conventional treatment remains suboptimal. Thus, complementary medicine gradually become a new treatment option for treating threatened miscarriage. Gushen Antai Pills **(**GAP), a classic prescription of Traditional Chinese medicine (TCM), has became a popular complementary therapy to conventional western medicine (dydrogesterone) in treating threatened miscarriage in recent years. However, a systematic summary and analysis for its therapeutic effects is lacking. This meta-analysis aimed to systematically evaluate the effectiveness and safety of Gushen Antai Pills combined with dydrogesterone in the treatment of threatened miscarriage.

**Methods:** A systematic search across seven electronic databases was conducted from inception to 17 September 2022. Studies were included if they were randomized controlled trials (RCTs) evaluating the effect of integrating Gushen Antai Pills and dydrogesterone in patients with threatened miscarriage, and reported the outcomes of interest. All statistical analyses were conducted using the Revman5.3 and Stata 13 software. The GRADE system was used to evaluate the quality of evidence.

**Results:** Ten eligible randomized controlled trials involving 950 participants were contained in this meta-analysis. The pooled analysis showed that Gushen Antai Pills combined with dydrogesterone can significantly reduce the incidence of early pregnancy loss (RR: 0.29; 95% CI: 0.19–0.42; *p* < 0*.*00001) and alleviate clinical symptoms (RR: 1.39; 95% CI: 1.22–1.59; *p* < 0*.*00001), compared with treatment of dydrogesterone alone. Also, meta-analysis indicated that integrating Gushen Antai Pills and dydrogesterone is more effective than using dydrogesterone alone in improving hormone levels (serum levels of progesterone, *β*-HCG and estradiol) for women with threatened miscarriage (all *p* < 0*.*00001). Meanwhile, the combined effects with significant heterogeneity also showed favorable consistency in the sensitivity analyses, indicating a good stability of present results. Moreover, no significant differences between Gushen Antai Pills combined with dydrogesterone and control group on adverse events was identified. The overall GRADE qualities were low to moderate.

**Conclusion:** The overall available evidence suggested that Gushen Antai Pills combined with dydrogesterone had significant effects in improving pregnancy success rate, clinical symptoms and hormone levels for women with threatened miscarriage, with considerable safety and reliability. However, due to the partial heterogeneity, suboptimal quality and high risk of bias of some included studies, further rigorously designed randomized controlled trials are required.

**Systematic Review Registration:** identifier https://INPLASY2022120035, https://inplasy.com/inplasy-2022-12-0035/.

## Introduction

Threatened miscarriage (TM) is a common complication of all pregnancies worldwide, occurring in approximately 20% in the first half of pregnancy (Lee et al., 2017). Unfortunately, owing to the complex pathogenesis and unsatisfied treatment in TM, nearly half of TM patients may progress to inevitable or incomplete miscarriage (Everett, 1997; Weiss et al., 2004). The miscarriage could bring physical and mental harms to the most of sufferers and their families, as well as a medical expense burden on patients’ families and society (Yang et al., 2013). Hence, identifying a prompt and effective treatment for TM is of significantly clinical and social importance (Sotiriadis et al., 2004).

Current evidence supposed that lacking progesterone due to the endocrine dysfunction is the most common factor (Ku et al., 2018; Dang et al., 2022). Progesterone is vital to maintain normal pregnancy in the first trimester, which enhances endogenous progesterone and chorionic gonadotropin secretion, as well as placental trophoblast activity (Chen et al., 2022). In this context, hormonal supplementation with progesterone was commonly used as the conventional western medicine for TM treatment, especially oral dydrogesterone (Coomarasamy et al., 2020). Dydrogesterone, pharmacologically comparable to endogenous progesterone, has highly selective progesterone action and good oral bioavailability and can be used at lower oral doses to exert curative effects (Omar et al., 2005). More importantly, dydrogesterone does not have an androgenic effect on fetuses and don't hinder progesterone synthesis in the placenta (Carp, 2015). With the wide use of dydrogesterone in treating TM, a number of studies had shown promising outcome that dydrogesterone can partially lower the incidence of subsequent miscarriage (Carp, 2012). However, dydrogesterone remedy also accompanied with some side effects such as nausea, headache, and sleepiness. Besides, solitary treatment with dydrogesterone rarely relieved all the symptoms of TM and yielded a limited therapeutic effect in some TM patients, especially those with multiple or complex causes (Lou et al., 2021). Presently, the clinical effect of dydrogesterone alone for treating TM remains suboptimal due to the considerable individual heterogeneity and poor prognosis of TM (Zhou et al., 2021). Thus, it is extremely essential to seek for more effective treatment options to treat TM. Recently, complementary and alternative medicine gradually becomes a new treatment option for treating TM. Especially, Traditional Chinese medicine (TCM) has became a popular complement to dydrogesterone in maintaining successful pregnancy preservation and the prevention of miscarriage in recent years (Chen et al., 2022).

According to the theory of TCM, the pathogenesis of TM included deficient kidney essence, deficiency of spleen-Qi, and liver depression (Ye et al., 2021; Zeng et al., 2021). Gushen Antai Pills (GAP), based on the treatment principles of tonifying kidney, invigorating spleen, and regulating liver, has been clinically applied for decades to treat TM (Ma et al., 2021). GAP included ten kinds of Chinese herbal medicines (Dipsaci, Cuscutae Semen, Herba Taxilli, Polygonum Multiflori, Rehmannia Glutinosa, Cistanche Deserticola, Atractylodes, Scutellariae, Paeoniae, and Uncaria), and each of its ingredients had respective therapeutical effects (Ma et al., 2021). Among the herbal formula, Dipsaci, Cuscutae Semen, and Herba Taxilli are the monarch drug, which play the role of tonifying the kidney, replenishing essence, nourishing blood, and consolidating Chong Vessel (Ye et al., 2021). On this basis, Rehmannia Glutinosa, Cistanche Deserticola, and Polygonum Multiflori were appended to boost the effects of strengthening kidney, invigorating spleen and liver, and nourishing blood (Ding et al., 2018; Ma et al., 2021; Chen et al., 2022). Meanwhile, Atractylodes, Uncaria, and Scutellariae are functional at Qi-invigorating, heat-clearing, harmonizing Qi, soothing liver, and detoxifying (Yang et al., 2021; Y. Xu, 2021, S.P. Ge, 2020). In brief, all the herbal components will be mutually reinforcing, achieving a more harmonious effect of tranquilizing the fetus to prevent miscarriage. Meanwhile, emerging clinical studies showed that applying dydrogesterone combined with GAP has a beneficial effect in the treatment of TM. However, a comprehensive and systematic assessment of this issue is lacking. Hence, this systematic review was conducted to comprehensively evaluate the efficacy and safety of integrating GAP and dydrogesterone for treating TM through summarizing available evidence, aiming to emphasize the therapeutic potential and feasibility of GAP in conjunction with dydrogesterone and highlight its safety and implications for practice in treating TM. Moreover, this topic will provide better evidence to guide the rational therapy in clinical practice and develop the new guidelines, further promoting the clinical treatment and understanding for TM.

## Materials and methods

This meta-analysis was performed according to the criteria of the PRISMA statement and the guidelines of the Cochrane Handbook for Systematic Reviews of Interventions (Higgins et al., 2011; Moher et al., 2015). This protocol has been registered in the INPLASY (https://inplasy.com/), with the registered number INPLASY2022120035.

### Literature search and strategy

A systematic search was conducted to identify relevant studies, which included seven databases of China National Knowledge Infrastructure (CNKI), the Chinese BioMedical database (CBM), Chinese Scientific Journal Database, PubMed, Cochrane Library, Embase, and the Wanfang database. The retrieval deadline was 17 September 2022. We applied a combination of free-text and medical subject heading (MeSH) terms to search (“Gushen Antai” AND “dydrogesterone” OR “duphaston” AND “threatened abortion” OR “threatened miscarriage”). Search strategy taking PubMed as an example were as follows: [threatened miscarriage OR threatened miscarriage (MeSH) OR threatened abortion (Title/Abstract) OR threatened miscarriage (Title/Abstract)] AND [dydrogesterone OR dydrogesterone (MeSH) OR dydrogesterone (Title/Abstract) OR duphaston (Title/Abstract)] AND [Gushen Antai OR Gushen Antai (MeSH) OR Gushen Antai (Title/Abstract)]. Furthermore, we also manually checked the references from the retrieved articles to seek the potential studies.

### Selection and data extraction

Two investigators (KC and XL) selected the studies and extract data from selected studies independently. Disagreements were resolved by group consensus. For literature with insufficient data, we will attempt to contact the corresponding author to obtain raw data. Firstly, the literatures retrieved from the initial searching was imported in the Endnote software and removed the duplication. Secondly, literature titles and abstracts were reviewed for further screening subsequently. Thirdly, full texts were screened in accordance with the inclusion and exclusion criteria. Inclusion criteria: 1) Participants: Pregnant women diagnosed with TM in the first trimester, regardless of underlying causes; 2) Intervention: Treatment with GAP combined with dydrogesterone; 3) Controls: Treated with dydrogesterone or progesterone alone; 4) Outcomes: The incidence of early pregnancy loss was the primary outcome. Secondary outcomes involved serum *β*-human chorionic gonadotropin (β-HCG), progesterone, and estradiol (E2) levels, as well as the rate of alleviation of clinical symptoms and adverse events (AEs); 5) Design: All randomized controlled trials (RCTs) were included. Exclusion criteria were listed as follows: I) Repetitive studies; II) Non-RCTs studies, retrospective studies, conferences, and reviews; III) Studies lacked the outcomes of interest or the valid data. The basic information were extracted from the included trails: study design, first author, published year, sample sizes of treatment and intervention groups, drug intervention strategy, treatment of control group, duration of treatment, main outcomes, and the modified Jadad score.

### Assessment of bias risk and quality of included studies

Two investigators (KC and XL) independently assessed the risk of bias of the included RCTs using the risk of bias tool of the Cochrane Handbook (Higgins et al., 2011). The next characteristics were evaluated: random (selection bias), allocation concealment, blinding (performance bias), incomplete outcome (attrition bias), reporting bias, and other possible biases. The modified Jadad score was also applied to assess the quality of all RCTs, ranging from 0 to 7 points. The 0 to 3 points stood for a low quality, while the RCTs with 4–7 points are named as a high quality (Jadad et al., 1996).

### Data synthesis and analysis

RevMan 5.3 (Nordic Cochrane Centre) and Stata 13.0 (Stata Corp LP) softwares were used for statistical analyses. Dichotomous data were expressed as relative risk (RR) with 95% confidence interval (CI) and continuous data were presented as Standardized mean differences (SMD) or mean differences (MD) with 95% CI. Descriptive approach would be used if the data were insufficient. Chi-squared test and the I^2^ statistic were used to assess the heterogeneity (I^2^ > 50% for a significant heterogeneity) (Higgins et al., 2003). Meta-analyses were conducted using fixed-effect or random-effect model (if I^2^ > 50%) where appropriate. If obvious heterogeneities were observed, sensitivity or subgroup analysis would be used to investigate potential sources of heterogeneity through sequentially omitting trials. Funnel plot and the Begg´s regression test were performed to check the publication bias. The trial sequential analysis (TSA) was used to decide whether sample sizes were adequate to evaluate outcomes of this meta-analysis (Gu et al., 2022). Two-sided *p* < 0.05 was deemed statistically significant.

### Assessment of evidence quality

Two independent reviewers (K. C and XX. L) assessed the quality of the evidence for each outcome using the GRADE approach (Guyatt et al., 2008). Divergences were discussed with and solved by the third reviewer. The evidence grade was classified as very low, low, moderate, or high based on the characteristics of study limitations, inconsistency, indirectness, imprecision, and reporting bias.

## Results

### Studies inclusion and assessment

#### Selection process of studies

**FIGURE 1:**
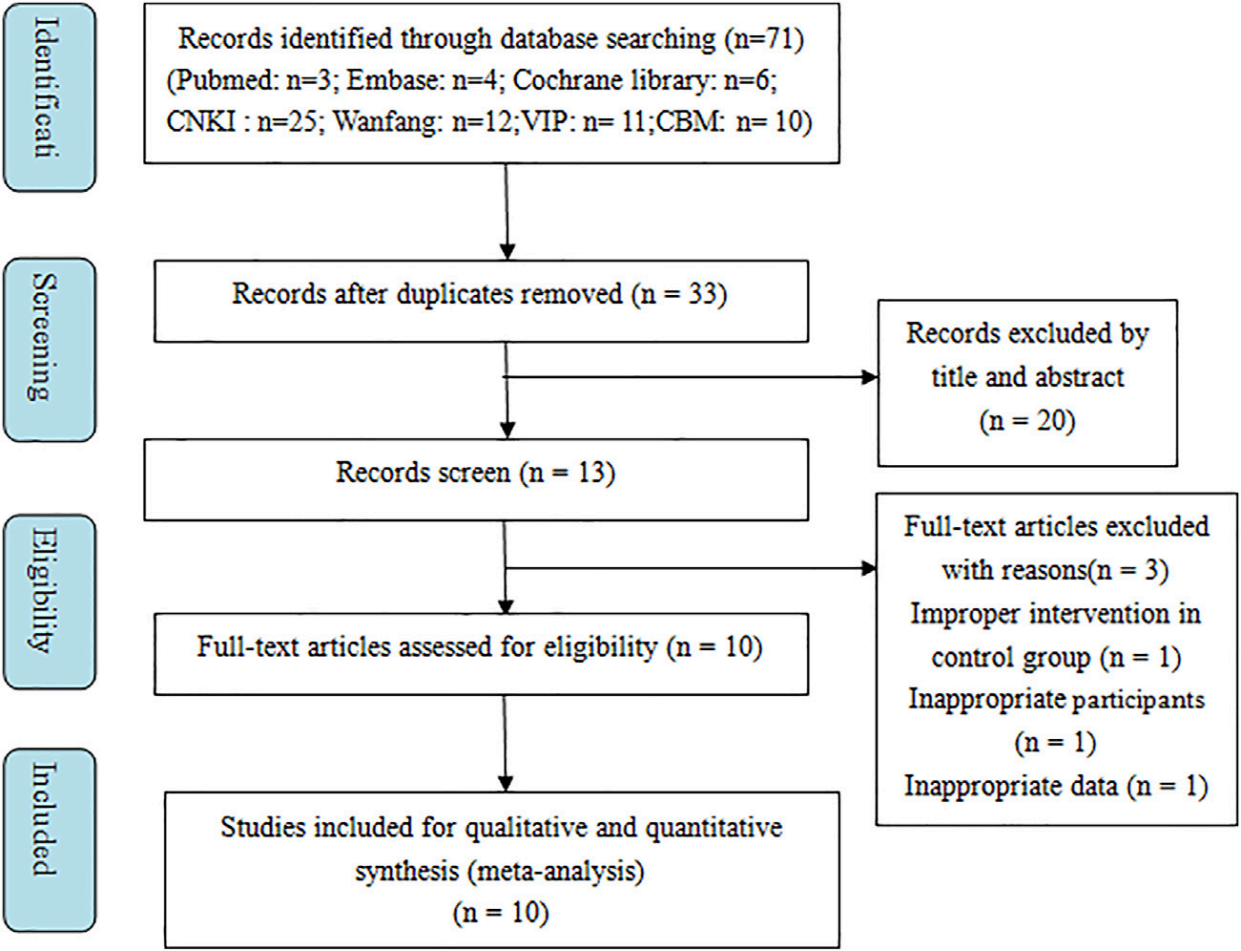
Flow chart indicates the selection process of this meta-analysis.

Initially, a total of 71 relevant studies were retrieved from the systemic search. 38 records were excluded for duplicates, then 33 records were filtered by screening the titles and abstracts. 20 records were eliminated for specific reasons according to the inclusion and exclusion criteria. Subsequently, the remaining 13 trails were assessed for eligibility, and 3 were further removed for inappropriate intervention, participants or data. Finally, 10 RCTs were included in this meta-analysis (X.Y. Zhang, 2020; Y. Gu, 2019; Huang and Xu, 2020; Y. Yang, 2020; S.P. Ge, 2020; Zhang and Qian, 2016; L.Y. Zhang et al., 2014; L.P. Li et al., 2018; Y. Xu, 2021; Y.Y. Lu et al., 2017). The selection process was summarized in Figure 1.

#### Study characteristics

The basic characteristics were shown in Table 1. All studies were published from 2014 to 2021 and came from China. Totally, this meta-analysis involved 950 participants, of which 495 participants were designated to the experimental group while 495 patients were assigned to the control group. Patients in the experimental group were treated with GAP in combination with dydrogesterone. The control group adopted the therapy of western medicine alone, among which nine trails used dydrogesterone and one with intramuscular injection of progesterone. Treatment durations were all 2 weeks. According to the assessment of the modified Jadad score, six RCTs were defined as low quality, while four RCTs were named as high quality.

**TABLE 1:** Basic characteristics of included literatures.

First author	Sample size	Average age (years)		Gestational age (Weeks)		Intervention strategy		Duration	Main outcomes	Jadad
(Publication year)	(E/C,N)	E	C	E	C	E	C			
Zhang. (2020)	50/50	29.82 ± 2.45	28.82 ± 2.94	8.35 ± 1.37	8.28 ± 1.81	GAP + Dydrogesterone	Dydrogesterone	2 weeks	①②③⑥	3
Gu. (2019)	35/35	30.6 ± 4.10	31.2 ± 4.40	7.2 ± 0.80	7.4 ± 0.90	GAP + Dydrogesterone	Dydrogesterone	2 weeks	①②③④⑤	5
Huang and Xu. (2020)	39/39	29.59 ± 2.05	29.51 ± 1.98	7.18 ± 0.77	7.21 ± 0.74	GAP + Dydrogesterone	Dydrogesterone	2 weeks	①②③④⑤⑥	5
Yang. (2020)	55/55	28.46 ± 3.55	28.99 ± 3.72	8.36 ± 1.11	8.45 ± 0.99	GAP + Dydrogesterone	Dydrogesterone	2 weeks	①②③	3
Ge. (2020)	52/52	30.18 ± 5.39	30.29 ± 5.14	9.45 ± 1.36	9.32 ± 1.24	GAP + Dydrogesterone	Dydrogesterone	2 weeks	①②③⑤	5
Zhang and Qian. (2016)	60/60	26.8 ± 1.30	26.2 ± 1.20	7.49 ± 0.31	7.33 ± 0.27	GAP + Dydrogesterone	Dydrogesterone	2 weeks	①②③④⑤	3
Zhang et al. (2014)	44/44	27.55 ± 4.47	27.68 ± 3.60	7.41 ± 0.87	7.64 ± 0.62	GAP + Dydrogesterone	Progesterone	2 weeks	①⑤⑥	3
Li et al. (2018)	50/50	27.87 ± 3.36	26.69 ± 3.45	7.66 ± 0.76	7.51 ± 0.77	GAP + Dydrogesterone	Dydrogesterone	2 weeks	①②③⑤	3
Xu. (2021)	40/40	27.7 ± 1.70	27.6 ± 1.60	6.0–12.0	6.0–12.0	GAP + Dydrogesterone	Dydrogesterone	2 weeks	①②③⑤	2
Lu et al. (2017)	50/50	27.0 ± 3.30	27.7 ± 2.90	8.63 ± 1.54	8.34 ± 1.51	GAP + Dydrogesterone	Dydrogesterone	2 weeks	①⑤	5

**Notes**: E, experimental group; C, control group; GAP, gushen antai pills; ①Incidence of early pregnancy loss; ②Serum progesterone level; ③Serum *β*-HCG, level; ④ Serum estradiol (E2) level; ⑤Alleviation of clinical symptoms; ⑥Incidence of adverse reactions.

#### Risk of bias assessment

**FIGURE 2:**
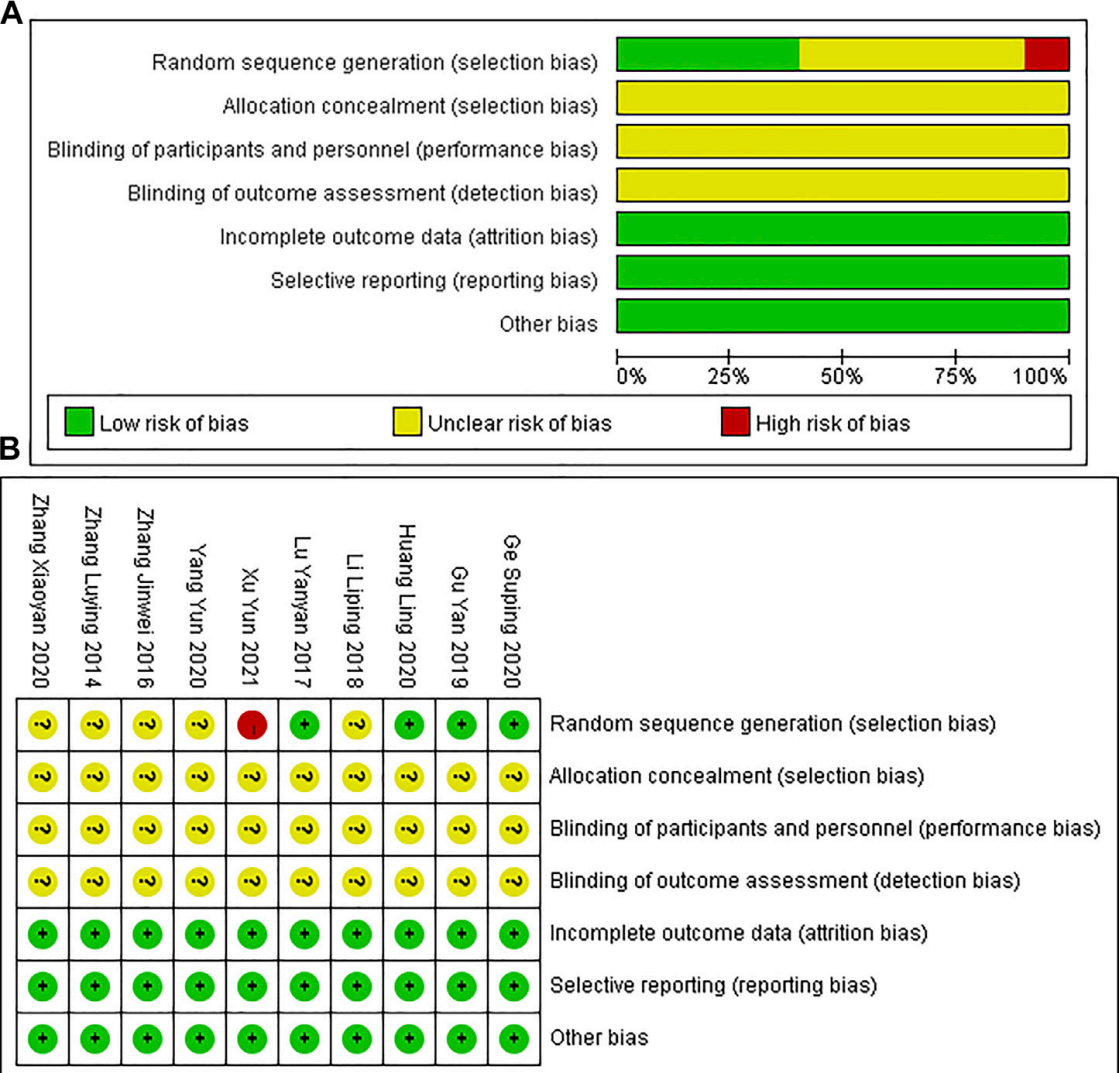
Risk-of-bias analysis: **(A)** Risk of bias graph; **(B)** Risk of bias summary.

Assessment of risk biases were displayed in Figure 2. In general, the overall methodological qualities of the included RCTs were poor to moderate. In terms of random sequence generation, four RCTs reported proper random methods and were designated as low risk; five trials supplied unclear randomization procedure, assessed as unclear risk; one trial did not mention the randomization, identified as a high risk. In terms of the allocation concealment and blinding assessment, all RCTs did not mention it clearly and were ranked as unclear risk. With respect to the incomplete outcome data, all included RCTs didn’t report the bias of selective reporting, thus assessing as low risk.

### Meta-analysis outcome

#### Early pregnancy loss

**FIGURE 3:**
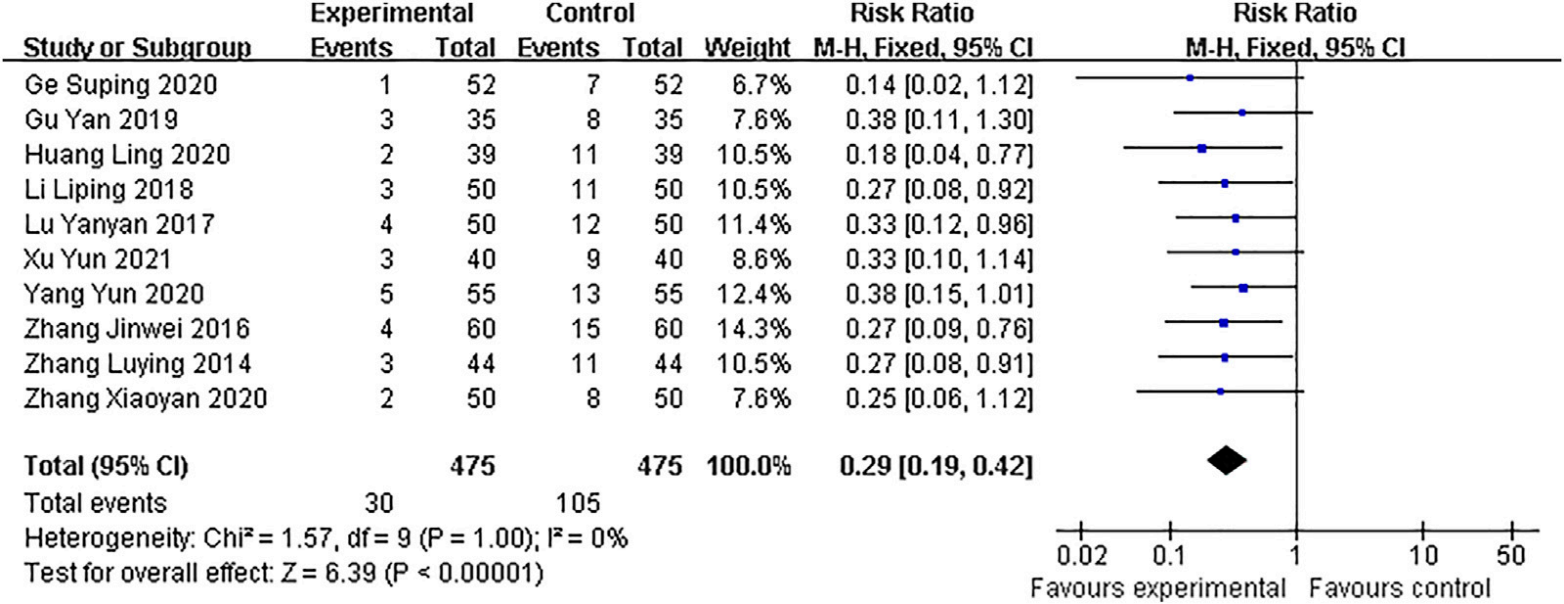
Forest plots assessing the outcomes of incidence of early pregnancy loss.

All of included RCTs stated the rate of early pregnancy loss. A fixed-effect model was adopted owing to the low heterogeneity between studies (I^2^ = 0%). Pooled analysis indicated that the GAP combined with dydrogesterone group had a significantly lower rate of early pregnancy loss when comparing with the dydrogesterone treatment alone (RR: 0.29; 95% CI: 0.19–0.42; *p* < 0*.*00001) (Figure 3).

#### Serum *β*-HCG levels

**FIGURE 4:**
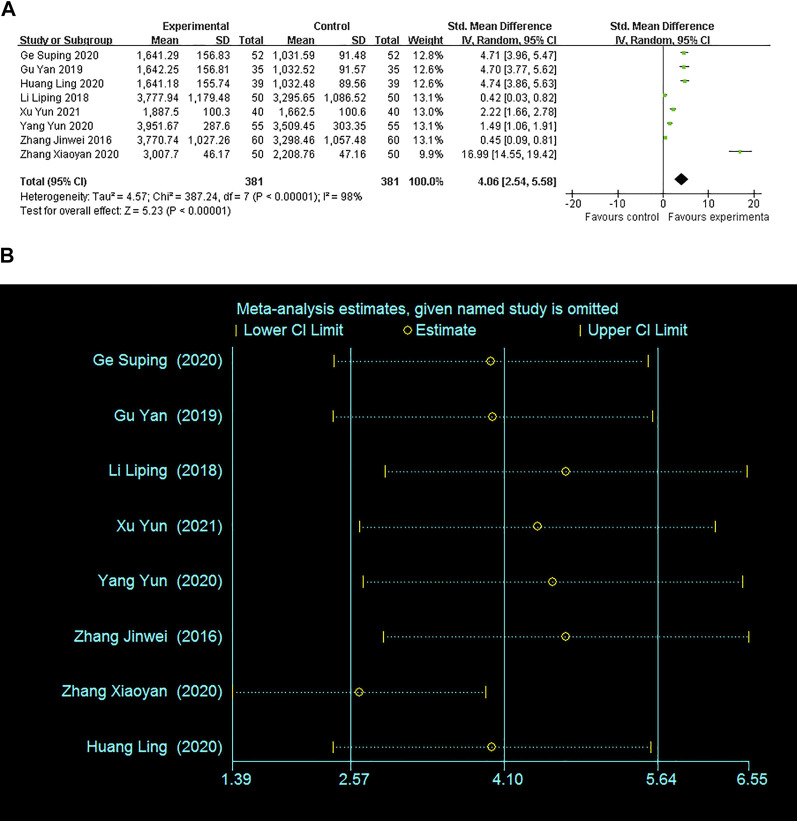
Forest plots with: **(A)**Serum *β*-HCG levels; **(B)**Sensitivity analysis for the *β*-HCG levels.

Eight studies comprising 762 patients compared the changes of serum *β*-HCG levels. There was obvious heterogeneity among these studies (I^2^ = 98%). The pooled result using a random effects model revealed that serum *β*-HCG levels in the experimental group were significantly higher after adjuvant treatment (SMD = 4.06; 95% CI: 2.54–5.58, *p* < 0.00001) (Figure 4A). The sensitivity analysis demonstrated a stable result with pooling outcomes ranging from SMD2.63 (95% CI: 1.38–3.88; I^2^ = 97%) to SMD4.67 (95% CI:2.87–6.48; I^2^ = 98%) (Figure 4B).

#### Serum progesterone levels

**FIGURE 5:**
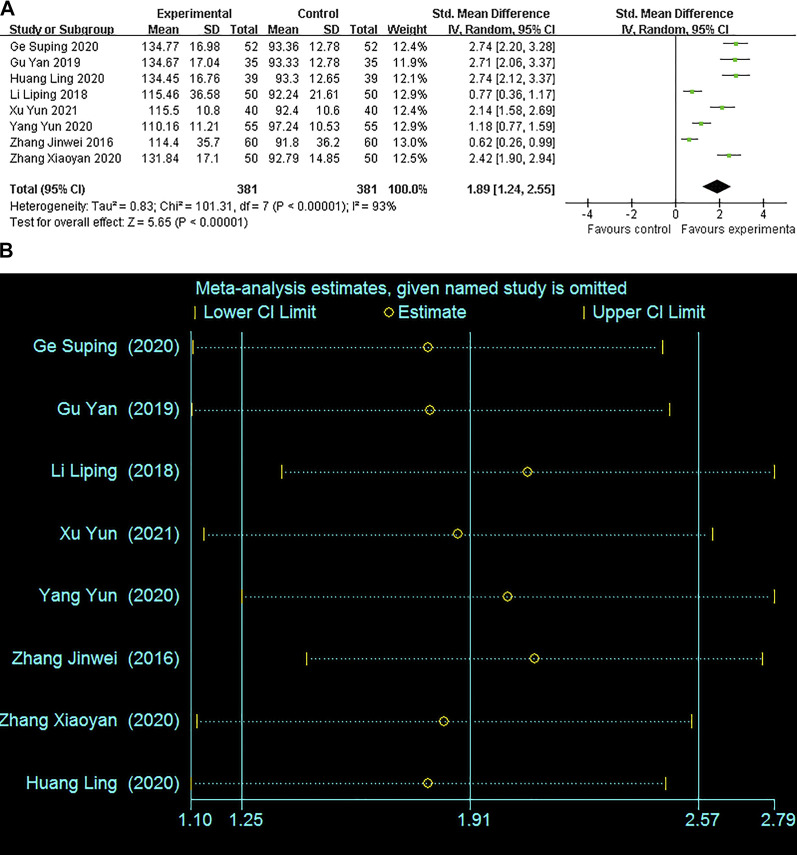
Forest plots with: **(A)** Serum progesterone levels; **(B)**Sensitivity analysis for the progesterone levels.

The serum progesterone levels were highlighted in 8 studies involving 762 patients. Random-effect model was applied owing to the significant heterogeneity (*I* ^*2*^ = 93%). The pooling result revealed that the serum progesterone levels in the combined treatment group were significantly higher after treatment (SMD = 1.89; 95% CI: 1.24–2.55, *p* < 0*.*00001) (Figure 5A). The sensitivity analysis suggested that single study could not obviously affect the pooled results, ranging from SMD1.77 (95% CI:1.10–2.44; I^2^ = 93%) to SMD2.08 (95% CI:1.42–2.73; I^2^ = 91%) (Figure 5B).

#### Serum E2 levels

**FIGURE 6:**
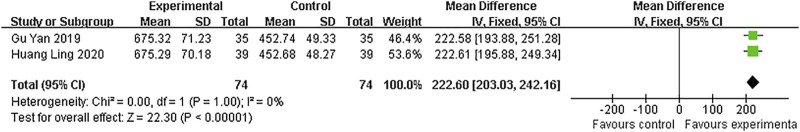
Forest plots with serum estradiol levels.

Only two studies reported serum E2 levels. The fixed-effect model was adopted to analyze based on the low heterogeneity (I^2^ = 0%). The pooled outcome indicated comparing with the control group, E2 has a significant improvement in the experimental group (MD = 222.60, 95% CI 203.03–242.16, *p* < 0*.*00001) as shown in Figure 6.

#### Alleviation of clinical symptoms (vaginal bleeding and abdominal pain)

**FIGURE 7:**
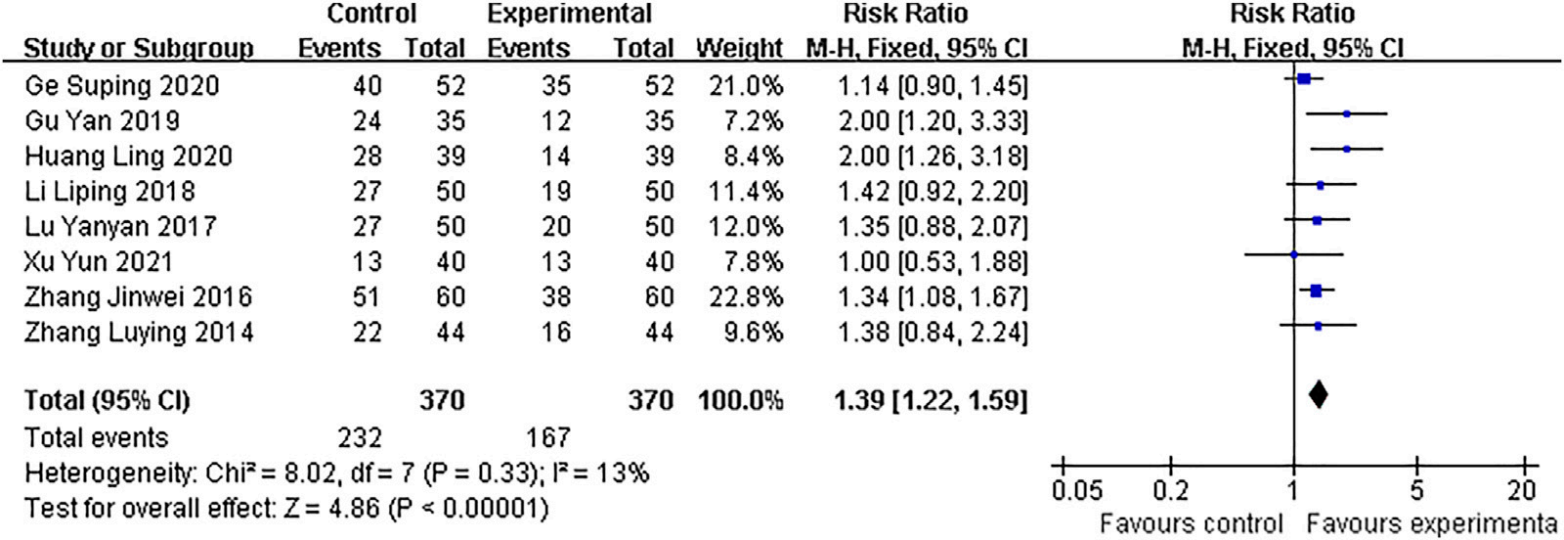
Forest plots with the alleviation of clinical symptoms.

Eight studies assessed the alleviation of clinical symptoms. The pooled result showed that combined medicines treatment was more effective in alleviating clinical symptoms than dydrogesterone treatment alone (RR: 1.39; 95% CI: 1.22–1.59; *p* < 0*.*00001), with low heterogeneity (I^2^ = 13%). (Figure 7).

#### Adverse events

**FIGURE 8:**
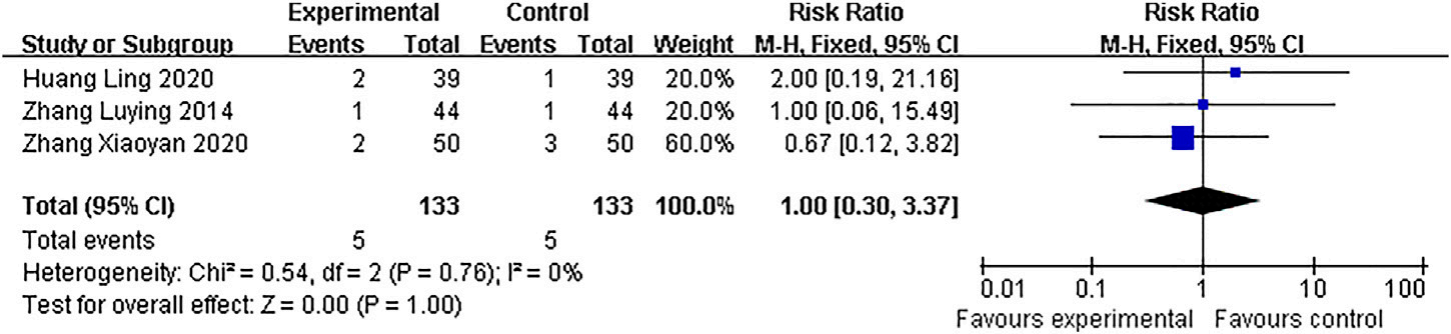
Forest plots with the incidence of adverse reactions.

Of the ten included studies, only three studies reported minor AEs in the treatment group, including one case of urticaria, two cases of nausea, and two cases of vomiting. However, the remaining seven studies lacked the information of AEs. Furthermore, based on the available data, the pooled analysis showed that AEs had no significant difference between 2 groups (RR = 1.0, 95% CI: 0.30–3.37, *p* = 1.00), without heterogeneity (I^2^ = 0%) (Figure 8).

#### Publication bias and sensitivity analysis

**FIGURE 9:**
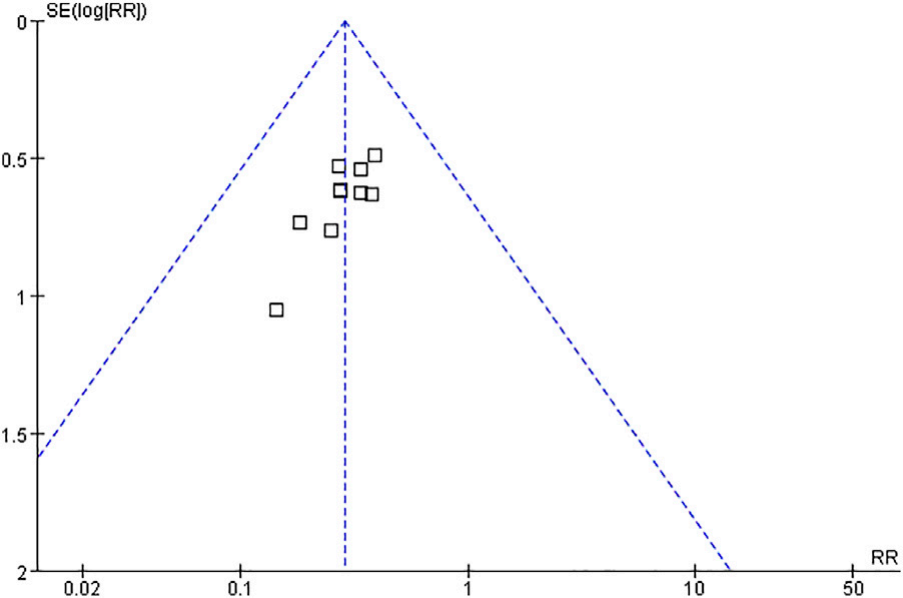
Funnel plot of the primary outcome.

Publication bias was expressed based on the data of the primary outcome. Although the funnel plot was asymmetrically distributed, the result of Begg’s test (*p* = 0.107) indicated no significant publication bias (Figure 9). All of sensitivity analysis results (including the evaluation of serum *β*-HCG and progesterone levels) remained stable, supporting the robustness of the pooled outcomes.

#### TSA assessment and quality of evidence

We used TSA to estimate the required sample size (greater than 117) for meta-analysis cumulative data, supporting the present sample size was adequately powered to evaluate outcomes*.* Briefly, the overall quality was moderate for the early pregnancy loss rate and alleviation rate of clinical symptoms; low for serum progesterone, *β*-HCG, E2 levels, and adverse events. The GRADE evaluations were displayed in Table 2.

**TABLE 2:** GRADE evidence profiles.

Outcomes	No. of participants (studies)	Relative effect (95% CI)	Absolute	Quality	Importance
Early pregnancy loss rate	950 (10 RCTs)	RR 0.29 (0.19–0.42)	157 fewer per 1,000 (128 fewer to 179 fewer)	⨁⨁⨁O^a^	CRITICAL
				Moderate	
Incidence of adverse events	266 (3 RCTs)	RR 1.0 (0.3–3.37)	0 fewer per 1,000 (26 fewer to 89 more)	⨁⨁OO^ac^	CRITICAL
				LOW	
Alleviation rate of clinical symptoms	740 (8 RCTs)	RR 1.39 (1.22–1.59)	176 more per 1,000 (99 more to 266 more)	⨁⨁⨁O^a^	IMPORTANT
				Moderate	
progesterone levels	762 (8 RCTs)	—	SMD 1.89 higher (1.24–2.55 higher)	⨁⨁OO^ab^ LOW	CRITICAL
β-HCG levels	762 (8 RCTs)	—	SMD 4.06 higher (2.54–5.58 higher)	⨁⨁OO^ab^ LOW	CRITICAL
Estradiol levels	148 (2 RCTs)	—	MD 222.6 higher (203.0–242.2higher)	⨁⨁OO ^ac^ LOW	IMPORTANT

**Note:** a, limitations (poor methodological quality); b, serious inconsistency (obvious heterogeneity); c, mild publication bias.

## Discussion

Currently, although great progress has been achieved in the understanding and treatment of TM, no definitely effective treatment is available for clinical management of TM. Growing studies have found that the therapeutic strategy of dydrogesterone combined with GAP has a potentially beneficial effect in treating TM. Herein, our study systematically integrated the existing RCTs and evaluated the efficacy and safety of GAP in conjunction with dydrogesterone in the treatment of TM, providing an evidence-based framework for further clinical application and research. Pooled results indicated that GAP combined with dydrogesterone can significantly decrease the rate of early pregnancy loss in pregnant women experiencing TM without increasing the incidence of AEs. Also, combined treatment was more efficient than dydrogesterone alone in relieving the clinical symptoms of TM, mainly including vaginal bleeding and abdominal pain. Furthermore, our meta-analysis showed that integrated GAP and dydrogesterone treatment was superior to dydrogesterone alone in improving serum progesterone, *β*-HCG and E2 levels.

TCM has unique treatment theories and practical experiences gradually formed in long-term medical practice, made indelible contributions in maintaining maternal and child health. Based on the TCM theory, TM belonged to the categories of fetal leakage with pathogenesis being commonly dominated by the kidney-Qi deficiency (Zeng et al., 2021). Moreover, other pathogenesis were also involved in the development of TM, including insufficiency of spleen-Qi, liver depression, and blood deficiency. Therefore, the rational treatment was focused on tonifying kidney, enhancing spleen, nourishing blood, as well as soothing liver (Li HF. et al., 2020a). GAP formula, adapted from the Shennong’s classic of materia medica and innovated by modern pharmaceutical technology, has a history-proven therapeutic efficacy (L.Y.Zhang et al., 2014). Among the herbal formula of GAP, Dipsaci, Cuscutae Semen and Herba Taxilli played the role of tonifying the kidney, nourishing blood, replenishing essence, and consolidating Chong Vessel (Ye et al., 2021). Moreover, Rehmannia Glutinosa, Cistanche Deserticola, and Polygonum Multiflori reinforced the effects of strengthening kidney, invigorating spleen and liver, and nourishing blood (Ding et al., 2018; Ma et al., 2021; Chen et al., 2022). In addition, Atractylodes, Uncaria, and Scutellariae would be beneficial to the Qi-invigorating and harmonizing, heat-clearing, soothing liver, and detoxifying (Yang et al., 2021, Y; Xu, 2021, S.P; Ge, 2020). Briefly, the herbal components of GAP can be mutually strengthened, yielding a more harmonious effect of tranquilizing the fetus to prevent miscarriage.

Evidence clarifying the effectiveness of GAP for TM can be identified in modern pharmacological studies. Holistically, pharmacological researches indicated that GAP benefited TM potentially through multiple mechanisms including enhance vascular function, improve immune activity, and regulate ovarian endocrine function, etc (Wang et al., 2021; Chen et al., 2022). In addition, modern pharmacological researches have pointed that baicalein, flavanone, norwogonin, rhynchophylline, polysaccharides, methoxyflavone, acacetin, anthraquinone glycosides and triterpenoid saponins are the main active components in GAP, which can improve blood circulation and placental blood supply, regulate disordered cellular immunity and humoral immunity, and improve endocrine dysfunction (Cao et al., 2020; Dang et al., 2022). For instance, baicalein and norwogonin can ameliorate the uterine blood circulation to nourish the embryo and strengthen the immunomodulation and inhibit allergic reactions to protect the fetus (Liao et al., 2021; Wu et al., 2022). Moreover, flavanone, acacetin, and methoxyflavone can suppress the formation of TNF-α through mitogen-activated protein kinase (MAPK) signaling pathway to attain the bactericidal and anti-inflammatory effects, maintaining the stabilization of intrauterine environment and reducing the miscarriage (Chang et al., 2017; Singh et al., 2021; Dang et al., 2022). Meanwhile, dydrogesterone also influence the cytokine balance and inhibit the immune activity at the fetal-maternal interface. As a result, GAP in conjunction with dydrogesterone can reinforce each other and reduce the occurrence of miscarriage (Y.Yang, 2020).

Recently, some pharmacological studies targeting the mechanisms of single herb or herb pair also provided indirect evidence for the anti-abortive effects of GAP. Wu HW found that Cuscutae Semen may prevent miscarriage by inhibiting the MAPK signaling pathway (Wu et al., 2018). Evidences have shown that during the implantation of embryo, the MAPK signal pathway will be activated and improve the invasion and proliferation of trophoblast cells and decidual stromal cells (DSCs), which play the key role in the maintenance of normal pregnancy (Paliga et al., 2005; Yang et al., 2020). Besides, it was believed that the Th1/Th2 imbalance and natural killer (NK) cell-regulated immunity significantly affect the development of miscarriage. More specifically, excessive expression of Th1 cytokines [interleukin-2 (IL-2), interferon-γ (IFN-γ), and tumor necrosis factor-*α* (TNF-α)] would lead to pregnancy failure, while low expression of Th2 cytokines (IL-4, IL-5, IL-6, IL-10, and IL-13) indicated a weak immunoprotective effect to maintain normal pregnancy (Calleja-Agius et al., 2011; Dong et al., 2017). Moreover, Zhong XH speculated that Scutellariae and Atractylodescan inhibited the maternal-fetal interface immunity and reduced the early embryo loss, which may be associated with its ability to the infiltration of NK cells and the production of IL-2, and upregulate the IL-10 in the uterus (Zhong et al., 2002; Zhong et al., 2008). Similarly, Ma AT showed that Scutellariae can play an anti-abortive effect by reducing the IFN-*γ* and increasing the progesterone (Ma et al., 2009).

Progress on network pharmacology provided some valuable clues about the molecular mechanisms of the holy herb pair Cuscutae Semen- Herba Taxilli (the main ingredients of GAP) in treating TM, which identified three key pathways directly related to TM, including MAPK, phosphatidylinositol-3-kinase/protein kinase B (PI3K-AKT), and transforming growth factor-*β* (TGF-*β*) (Yang et al., 2020). Identically, both PI3K-AKT and TGF-β signaling pathways participates in the proliferation, migration and apoptosis of trophoblast cells that are vital for establishing and persisting normal pregnancy (Latifi et al., 2019). Additionally, the TGF-β signal pathway is also involved in immune and inflammatory responses to promote immune tolerance of the maternal-fetal interface, maintaining the fetus in a stable state (Liu et al., 2017; Yang et al., 2020).

Network pharmacological analysis further found some valuable genes and signaling pathway of GAP for treating TM, including AKT 1, vascular endothelial growth factor A (VEGFA), Signal transducer and activator of transcription 3 (STAT3), and hypoxia-inducible factor 1(HIF-1) signaling pathway (Dang et al., 2022). AKT1, a heterodimer of AKT, mediates many PI3K-regulated downstream pathways (Gillani et al., 2021). Also, PI3K-AKT pathway can promote the growth of trophoblast cells and regulate the function of chorionic trophoblast cells, thus exerting a prominent effect on the embryonic development and pregnancy maintenance (Zhu et al., 2013). VEGFA, an important member of the VEGF family, can positively increase the vascular permeability and promote the formation of endometrial microvasculature and placenta through the upregulated expression AKT signaling pathway, thereby promoting embryonic growth and development (Amirchaghmaghi et al., 2015; Li Y. et al., 2020b).

As the research developed, it was shown that inhibiting STAT3 pathway expression will decrease the Cyclin D1 expression (Tang et al., 2018). Relevant studies have also confirmed that the downregulation of Cyclin D1 can decreased the number of trophoblast cells and increased the cell apoptosis, thus affecting the proliferation and differentiation of endometrial and trophoblast cells in first-trimester pregnancy (Cai et al., 2016). In addition, the inhibition of STAT3 also influences the VEGF expression and Th17 cells differentiation, and decrease the levels of related Th1/Th2 cytokines, resulting in immune imbalance and reduced angiogenesis, which affects the stability of the intrauterine immune environment and embryonic development (Lee et al., 2016). HIF-1 was also involved in the early miscarriage, which affect the receptivity of endometrium to the embryo. The overexpression of HIF-1 protein will cause endometrium to be infiltrated by inflammatory cells, thereby making the trophoblast cells difficult to invade the uterine spiral artery and reducing the placental blood perfusion, which lead to the placental angiogenesis deficiency and embryonic ischemia and hypoxia (Zhi et al., 2018; Colson et al., 2020; Zhao et al., 2021). Meaningfully, the Scutellariae-Atractylodes herb pair of GAP can rationally regulate the aforementioned target genes and signaling pathway to maintain stable pregnancy and prevent miscarriage (Dang et al., 2022). Although several mechanisms have been proposed, the precise mechanism of complementary GAP-treated TM has not fully elucidated owing to the multi-components and multi-effects of GAP. More researches involving *in vitro* and *in vivo* are needed to uncover the detailed mechanism.

The present results also documented that GAP combined with dydrogesterone can effectively improve endocrine levels of serum P, E2, and *β*-HCG. Remarkably, it’s well known that sex hormones of P, E2, and *β*-HCG play a crucial role in maintaining the maternal-fetal response and stability of the internal environment (Cha et al., 2012). Patients with TM usually had lower levels of serum P, *β*-HCG, and E2, which increased the risk of early pregnancy loss and miscarriage (Osmanağaoğlu et al., 2010). Therefore, improving serum hormone levels highly associated with the progression of TM is of great significance. As expected, modern pharmacological studies provided evidence that GAP can improve the sex hormones through a multiple-target pathway and holistic effect, especially the estrogen-like effect (Cao et al., 2020). Likewise, pharmacological studies have reported that Dipsaci and Cuscutae Semen could regulate the immune balance of maternal-fetal interface and the expression of Th2 cytokines to stabilize pregnancy, which also attributed to its estrogen-like activities (Yang et al., 2021). In addition, mounting studies had shown that Cuscutae Semen and some active compounds in the GAP could improve the secretion of *β*-HCG, which promote the generation of progesterone and secretion of estrogen, and inhibit the production of oxytocin to decrease the intensity of uterine contractions (Ma et al., 2021; Maharajan et al., 2021; Ye et al., 2021; Chen et al., 2022). Generally, dydrogesterone itself can replenish the inadequate endogenous progesterone (Carp, 2012). Furthermore, the application of GAP collaborate with dydrogesterone tend to yield a more harmonious and enhanced effect on regulating the relevant sex hormone levels (Y.Gu, 2019). Additionally, the present study also showed that GAP combined with dydrogesterone is more effective in reliving the clinical symptoms of pain and bleeding of TM patients, whose mechanisms are possibly related to exert estrogen-like effects, adjust the body’s immunity and endocrine disorders, and improve the intrauterine environment (Zhang and Qian, 2016; Zhang, 2020). However, improving the curative outcome of TM is a long-term and complex process and need systematic pharmacological examinations to fully verify.

Meanwhile, the safety assessment is also a concern in the clinical application of TCM. Based on the available data, our study showed that there were no obvious AEs with the combined treatment, indicating that GAP in conjunction with dydrogesterone may be safe and well-tolerated. In particular, GAP was processed through international quality control of natural medicinal herbs and permitted by the Chinese State Food and Drug Administration, which make it to be more rationalizing and standardizing (Ma et al., 2021). Although GAP has the characteristics of multiple herbs, optimized compatibility will decrease the side effects of the other herbs (Liu et al., 2015). However, it must be noted that most of the include trials did not report the data of AEs. It may be because the incidence of AEs was indeed too rare or ignore the awareness on the AEs. Presently, the systematic data regarding the potential harm to the mothers and fetuses in the utilization of GAP is insufficient. In the near future, we hope that detailed pharmacotoxicity studies assessing the AEs of GAP will be conducted, providing sufficient evidence of safety.

To our knowledge, this is the first meta-analysis to quantitatively estimate the beneficial effect of GAP adjuvant therapy for TM, providing better evidence-based evaluation for the domestic and international researchers to understand this issue. Moreover, most of the pooled results had low heterogeneity and were generally consistent with the sensitivity analyses, showing a good stability. Additionally, the current quality of overall evidence assessed by GRADE can offer a comprehensive and transparent framework for promoting clinical practice guidelines. Importantly, extensive and in-depth data mining in applying TCM to the treatment of TM is conducive to inherit the clinical experience of ancient TCM, boost the understanding of the theory and practice of TCM in TM treatment and enrich the treatment options for treating TM.

Despite our critical evaluation of the currently available evidence, some potential limitations should not be ignored. Firstly, limitations included the low quality of some of included RCTs owing to the unclear allocation concealment and attrition bias, reducing the strength of evidence. Therefore, the present results should be interpreted with cautious owing to the aforementioned limitations and further high-quality, well-designed RCTs on this theme are urgently needed. Secondly, Notwithstanding we performed a thoroughgoing literature search with no language restriction, all included trails were entirely conducted in China, which likely limits their representativeness and generalizability. Thirdly, very few trials included have reported the outcomes of serum E2 levels and AEs, thus the objective influence to E2 value and safety must be evaluated by more rigorous RCTs. Additionally, although no obvious heterogeneity existed across the studies, some confounding factors (age and miscarriage history) affecting the pooled results could not fully adjusted based on the present information.

## Conclusion

This systematic review provided evidence to the clinical practice that GAP combined with dydrogesterone may be effective and safe for treating patients with TM. However, the present results should be carefully interpreted because of the partial heterogeneity, suboptimal quality and high risk of bias of some included studies. In the future, further high-quality RCTs are required to ensure reliable effects of GAP adjuvant therapy for TM.

## Data Availability

The original contributions presented in the study are included in the article/Supplementary Material, further inquiries can be directed to the corresponding author.
